# Effect of single nucleotide polymorphisms on intramuscular fat content in Hungarian Simmental cattle

**DOI:** 10.5713/ajas.17.0773

**Published:** 2018-03-13

**Authors:** István Anton, Balázs Húth, Imre Füller, László Rózsa, Gabriella Holló, Attila Zsolnai

**Affiliations:** 1 NARIC-Research Institute for Animal Breeding Nutrition and Meat Science, Gesztenyes u. 1., Herceghalom, 2053, Hungary; 2 Department of Animal Husbandry and Management, Institute of Animal Science, Faculty of Agricultural and Environmental Sciences, University of Kaposvár, Kaposvár, Guba S. u. 40., 7400, Hungary; 3 Association of Hungarian Simmental Cattle Breeders, Zrinyi u. 3., Bonyhád, 7150, Hungary

**Keywords:** Cattle, Single Nucleotide Polymorphism, Genome-wide Association Study (GWAS), Fat

## Abstract

**Objective:**

To estimate effect of single nucleotide polymorphisms on the intramuscular fat content (IMF) of Hungarian Simmental bulls.

**Methods:**

Genotypes were determined on high-density Illumina Bovine DNA Chip. After slaughtering of animals, chemical percentage of intramuscular fat was determined from *longissimus dorsi* muscle. A multi-locus mixed-model was applied for statistical analyses.

**Results:**

Analyses revealed four loci (rs43284251, rs109210955, rs41630030, and rs41642251) to be highly associated (−log_10_P>12) with IMF located on chromosome 1, 6, 13, and 17, respectively. The frequency of their minor alleles was 0.426, 0.221, 0.162, and 0.106.

**Conclusion:**

The loci above can be useful in selection programs and gives the possibility to assist selection by molecular tools.

## INTRODUCTION

The main aim of modern livestock agriculture is to satisfy the growing demands of consumers for high-quality, safe and delicious animal products. This trend is evident for the beef industry as well. The proper breeding scheme in the past was very successful in increasing of lean meat content, but resulted in reduced intramuscular fat content (IMF) and changed muscle fibre types as well [1]. Nowadays, the main aim of numerous studies is improvement of IMF in beef [2] and investigation of relationship between beef quality and muscle biochemical characteristics such as connective tissue and muscle fibres [3]. The Association of Hungarian Simmental Cattle Breeders has been taking measures in the last years to improve beef quality. For breeding purpose and market expectations, it was developed a new dual purpose index where weighting of breeding values is quoted as follows: 40% for milk, 30% for meat and 30% for fitness [4]. At present, there is considerable interest in the application of genomic breeding value estimation to promote rapid and efficient selection in farm animals. Some single nucleotide polymorphisms (SNPs) have been demonstrated to affect IMF and meat quality traits in farm animals [5,6]. Genome-wide association study (GWAS) based on typing of 777,000 SNPs by DNA chip technique is suitable for the improvement of beef quality and IMF in different cattle breeds. Availability of genomic information on a large number of animals has changed the dairy cattle breeding worldwide [7]. In case of beef cattle [8,9] and dual purpose cattle, result of GWAS can also change the selection progress, though the effective population number and the accuracy of estimated genomic breeding value is lower compared to dairy cattle. Aim of present investigation was to estimate the effect of SNP polymorphisms on IMF.

## MATERIALS AND METHODS

A total of 146 Hungarian Simmental muscle samples from eleven farms were collected and were stored in liquid nitrogen at −196°C until DNA extraction. Animals were kept in identical conditions, fed the same diet and slaughtered at approximately similar live weight (530.6±44.7 kg), under commercial conditions using Hungarian standard procedure.

During slaughtering, rib samples have been taken from *longissimus dorsi* muscle (LD) cut between the 11th to 13th rib. Chemical percentage IMF was determined from LD. After removing surface fat, lipid content of LD was determined gravimetrically by Soxhlet method, using petroleum ether as solvent [10]. Lipid content (IMF values) ranged from 0.5% to 5.8%.

For isolation of genomic DNA from muscle, samples were incubated in 5% Chelex suspension and 0.6 μg/μL Proteinase-K solution at 56°C, overnight. Final treatment of the mixture was an incubating step at 98°C for ten minutes. After DNA extraction from LD, SNP typing was performed with high-resolution SNP chips developed for cattle (Illumina Bovine HD Chip, San Diego, CA, USA). Illumina Final Report file was imported into SVS software (GoldenHelix, Bozeman, MT, USA). Samples were excluded from analysis if call rate was below 95%. Only SNPs having consistently high call rate (>95%) were included in this study. Duplicated samples (IBD >0.95) were excluded from dataset. After excluding monomorphic loci and loci with minor allele frequency <0.05, dataset included 129 animals and 120,774 SNPs. Statistical analyses were performed by SVS software (GoldenHelix, USA). For visualising results, high resolution image has been produced by R using qqman library [11].

For correction of population structure, genomic kinship matrix has been used in multi-locus mixed-model [12]. Phenotypic values have been left as they were, a continuous variable.

The used model was

y=Xβ+Zu+e

Where y is the IMF, X is the matrix of fixed effects composed of SNPs and covariates (age and sex), Z is the matrix of random animal effects. The e means the residual effects. The β and u are vectors representing coefficients of fixed and random effects, respectively.

The Ensemble cow UMD3.1 and gene ontology (GO) [13] databases were used to look for surrounding of most significant SNPs and for functional categories.

## RESULTS AND DISCUSSION

**Figure 1:**
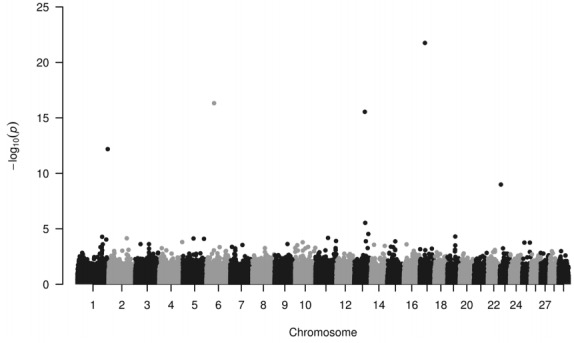
Manhattan plot of single nucleotide polymorphisms regarding intramuscular fat content (IMF). Loci on chromosome 1, 6, 13, and 17 display the highest −log_10_P values (see dots >12), which are associated with the intramuscular fat content in Hungarian Simmental cattle.

Four loci have been identified to be highly associated with IMF (Figure 1). These loci (−log_10_P>12) seem to be useful in selection programs and are located on chromosome 1, 6, 13, and 17, their minor allele frequencies are 0.426, 0.221, 0.162, and 0.106, respectively (Table 1). IMF values—extracted from the database consisting of recorded values for different metabolites and for carcass parts—ranged from 0.5% to 5.8%.

On chromosome 1 there are several protein coding sequences in near vicinity of the locus rs43284251, including polypeptide N-acetylgalactosaminyltransferase 15 (*GALNT15*), diphthamide biosynthesis 3 (*DPH3*), and serine/threonine-protein phosphatase 6 regulatory ankyrin repeat subunit A (*ANKRD28*).

*GALNT15* (GO:0030133, 0000139, 0016021, 0006486, 0030246, 0016757)—also known as *GALNTL2*, *pp-GalNAc-T15*, or *GALNT7*—is part of glycoproteome playing role in cell interactions with environment [14]. *GALNTs* are localised in Golgi and endoplasmatic reticulum (ER). Their traffic from Golgi to ER often activated in human malignant tumors. In human, nonhuman primates and rodents, loss of function of GALNT2 has been shown to lower high-density lipoproteins and species specific glycosylation targets has been identified [15]. An allele of GALNT13 in human has been found to be overrepresented in elite sprinters [16] suggesting involvement in energy pathways. Functions of GALNTs are not fully understood, but their targets [17,18] may affect lipoprotein metabolism and fat deposition in intramuscular space.

*DPH3* (GO:0046872, 0017183, 0051099, 0050709, 0005829, 0005654) is essential in the synthesis of diphtamide post-translationally modificate histidine elongation factor 2 [19]. Down-regulation increases extracellular release of proteoglycans, indicating a possible role in the secretion process.

ANKRD28 is related to pathways (http://pathcards.genecards.org/) like Vesicle-mediated transport and Transport to the Golgi and subsequent modification.

On chromosome 6 there are several protein coding sequences in near vicinity of the locus rs109210955, including leucine aminopeptidase (*LAP3*), mediator of RNA polymerase II transcription subunit 28 (*MED28*), family with sequence similarity 184 member B (*FAM184B*), DDB1 and CUL4 associated factor 16 (*DCAF16*), non-SMC condensin I complex subunit G (*NCAPG*), ligand-dependent nuclear receptor corepressor-like protein (*LCORL*). Haplotypes of *LAP3* (GO: 0008235, 0004177, 0030145, 0008233, 0097718, 0006508, 0070062, 0005925, 0005737, 0005739, 0005829, 0030496, 0005654) gene is reported [20] to be used as molecular marker in association with milk production and other performance related traits. *MED28* (GO:0019827, 0051151, 0016592, 0030864) has negative regulation effect on smooth muscle cell differentiation. *In vivo* fate mapping showed, that a set of adipocytes arose from smooth muscle-like origin [21]. *DCAF16* (GO:0016567, 0080008) is associated with average daily gain in cattle [22].

*NCAPG* (GO: 0007076, 0000793, 0005737, 0000779, 0000796) is a major player in cellular maintenance processes and a major contributor to genetic variability in bovine feed efficiency [23]. Expression of *NCAPG* together with *LCORL* (GO:0003677, 0006366, 0006357) measured in adipose and muscle tissue are associated with cattle feed intake and weight gain [24]. *NCAPG* affects arginine metabolism having influence on body-mass gain and lipid deposition.

Locus rs41630030 on Chr 13 is located close to ADP-ribosylation factor-related protein 1 (*ARFRP1*), TNF receptor superfamily member 6b (*TNFRSF6B ENSBTAG00000027407*). ARFRP1 (GO:0005525, 0007264, 0034067, 0043001, 0042147, 0007369, 0005802, 0016020, 0005829) is required for the lipidation of chylomicrons in the intestine and required for very low density lipoprotein (VLDL) lipidation in the liver [25], *TNFRSF6B* might have role in response to lipopolysaccharide according to one of the GO annotations (GO:0032496). Its expression has been detected in bovine ovarian follicles [26]. There are also two transcripts overlapping rs41630030; *ENSBTAT00000045108. 3* (GO:0005524, 0004003, 0032508) and *ENSBTAT00000052120.2.* (GO:0003677, 0005524, 0004003, 0032508, 0006139, 0005634). GO annotation number 0006139 suggests this transcript is involved in nucleobase-containing compound metabolic process.

Nearby locus rs41642251 on Chr 17 preferentially expressed antigen in melanoma (*PRAME*) and splicesomal RNA (U1) loci are located. *PRAME* (GO:0045892, 0045596, 0043066, 0008284) were shown that lipopolysaccharide together with type 2 interferon has increased its expression [27]. Literature on U1 was not available, however lack of activity of splicing factor SRSF10 affected development of adipose tissue [28] in cell culture. Contribution of different splicing events to the adipose tissue thermogenesis is involved in development of diet-induced obesity in mice [29].

Fatty acid binding protein 4 (*FABP4*) alleles in Japanese Black [30] and in Holstein cattle [31] have effect on IMF, whereas in Korean cattle [32] it affects backfat thickness. In Fleckvieh cattle [33] looked for genomic regions showing associations with IMF, analysing diacylglycerol o-acyltransferase 1 (*DGAT1*), *FABP4*, fatty acid synthase (*FASN*) and peroxisome proliferator activated receptor gamma coactivator 1 alpha (*PPARGC1A*) genes on chromosome 14, 14, 19, and 6, respectively. In our study, none of them was significantly associated with IMF content. However in previous studies we demonstrated the effect of leptin (Chr 4), *DGAT1* (Chr 14) and thyroglobulin (Chr 14) gene polymorphisms on the IMF of Angus cattle [34].

The IMF or marbling represents a valuable beef quality trait and is important determinant of palatability due to its contribution to juiciness and flavour. Meat with low IMF may be dry and flavourless. Higher levels of IMF have been associated with increased tenderness, juiciness and flavour of beef [35,36]. Molecular tests can provide facilities for direct selection among variants. However, benefits of different alleles depend on economic reasons given in the breeding programs. Herewith we propose to follow up segregation of alleles of the found loci in the Simmental population, and to continue phenotypic characterisations in the upcoming generations. After successful validation, selection for favourable alleles at reported loci on chromosomes mentioned above (Chr 1, 6, 13, and 17) could be utilised, if increased IMF is desirable.

## Figures and Tables

**Table 1:** List of loci associated with intramuscular fat content (IMF), their genomic location and nearest genes

Marker ss ID (variant type)	Chr	Position	−log_10_P	−log_10_P after Bonferroni correction	Nearest genes (their distance from the marker in bp)	MAF	FDR
rs43284251 intergenic	1	154894091	12.2	7.1	GALNT15 (29068), DPH3 (110416), ANKRD28 (325755)	0.426	2.6e-8
rs109210955 intergenic	6	39358026	16.3	11.2	LAP3 (757999), MED28 (749185), FAM184B (685720), DCAF16 (603129), NCAPG (545975), LCORL (365914)	0.221	2.4e-12
rs41630030 intronic	13	54540476	15.5	10.4	ARFRP1 (14722), TNFRSF6B (12525), ENSBTAT00000045108, ENSBTAT00000052120	0.162	1.3e-11
rs41642251 intergenic	17	26689850	21.7	16.7	PRAME (1567997), U1 (251068),	0.106	2.6e-17

MAF, minor allele frequency; FDR, false discovery rate; *GALNT15*, polypeptide N-acetylgalactosaminyltransferase 15; *LAP3*, leucine aminopeptidase; *MED28*, mediator of RNA polymerase II transcription subunit 28; *FAM184B*, family with sequence similarity 184 member B; *DCAF16*, DDB1 and CUL4 associated factor 16; *NCAPG*, non-SMC condensin I complex subunit G; *LCORL*, ligand-dependent nuclear receptor corepressor-like protein; *ARFRP1*, ADP-ribosylation factor-related protein 1; *TNFRSF6B*, TNF receptor superfamily member 6b; *PRAME*, preferentially expressed antigen in melanoma; *U1*, splicesomal RNA.

## References

[1] Hocquette JF, Gondret F, Baéza E (2010). Intramuscular fat content in meat-producing animals: development, genetic and nutritional control, and identification of putative markers. Animal.

[2] Mir PS, Schwartzkopf-Genswein KS, Entz T (2008). Effect of a short duration feed withdrawal followed by full feeding on marbling fat in beef carcasses. Livest Sci.

[3] Jurie C, Picard B, Hocquette JF (2007). Muscle and meat quality characteristics of Holstein and Salers cull cows. Meat Sci.

[4] Húth B, Holló I, Füller I, Polgár JP, Komlósi I (2013). Breeding and selection strategy in Hungarian Simmental cattle. Hungarian J Anim Prod.

[5] Anton I, Kovács K, Fésüs L (2008). Effect of DGAT1 and TG gene polymorphism on intramuscular fat and milk production traits in different cattle breeds in Hungary. Acta Vet Hung.

[6] Anton I, Zsolnai A, Holló I, Repa I, Holló G (2013). Effect of thyroglobulin gene polymorphism on the intramuscular fat content in cattle examined by x-ray computed tomography and Soxhlet methods. Arch Anim Breed.

[7] Nayeri S, Sargolzaei M, Abo-Ismail MK (2016). Genome-wide association for milk production and female fertility traits in Canadian dairy Holstein cattle. BMC Genet.

[8] Allais S, Leveziel H, Hocquette JF (2014). Fine mapping of quantitative trait loci underlying sensory meat quality traits in three French beef cattle breeds. J Anim Sci.

[9] Picard B, Jurie C, Duris MP, Renand G (2006). Consequences of selection for higher growth rate on muscle fibre development in cattle. Livest Sci.

[10] Soxhlet F (1879). Die gewichtsanalytische Bestimmung des Milchfettes. Dinglers Polytech J.

[11] Turner SD qqman: an R package for visualizing GWAS results using Q-Q and manhattan plots.

[12] Segura V, Vihjalmsson BJ, Platt A (2012). An efficient multi-locus mixed-model approach for genome-wide association studies in structured populations. Nat Genet.

[13] Ashburner M, Ball CA, Blake JA (2000). Gene ontology: tool for the unification of biology. The Gene Ontology Consortium. Nat Genet.

[14] Bard F, Chia J (2016). Cracking the glycome encoder: signaling, trafficking, and glycosylation. Trends Cell Biol.

[15] Khetarpal SA, Schjoldager KT, Christoffersen C (2016). Loss of function of GALNT2 lowers high-density lipoproteins in humans, nonhuman primates, and rodents. Cell Metab.

[16] Wang G, Padmanabhan S, Miyamoto-Mikami E (2014). GWAS of elite Jamaican, African American and Japanese sprint athletes. Med Sci Sports Exerc.

[17] Schjoldager KT, Vester-Christensen MB, Bennett EP (2010). O-glycosylation modulates proprotein convertase activation of angiopoietin-like protein 3: possible role of polypeptide GalNAc-transferase-2 in regulation of concentrations of plasma lipids. J Biol Chem.

[18] Schjoldager KT, Vakhrushev SY, Kong Y (2012). Probing isoform-specific functions of polypeptide GalNActransferases using zinc finger nuclease glycoengineered. SimpleCells. Proc Natl Acad Sci USA.

[19] Sjoelinder M, Uhlmann J, Ponstingl H (2004). Characterisation of an evolutionary conserved protein interacting with the putative guanine nucleotide exchange factor DelGEF and modulating secretion. Exp Cell Res.

[20] Zheng X, Ju Z, Wang J (2011). Single nucleotide polymorphisms, haplotypes and combined genotypes of LAP3 gene in bovine and their association with milk production traits. Mol Biol Rep.

[21] Long JZ, Svensson KJ, Tsai L (2014). A smooth muscle-like origin for beige adipocytes. Cell Metab.

[22] Zhang W, Li J, Guo Y (2016). Multi-strategy genome-wide association studies identify the DCAF16-NCAPG region as a susceptibility locus for average daily gain in cattle. Sci Rep.

[23] Widmann P, Reverter A, Weikard R (2015). Systems biology analysis merging phenotype, metabolomic and genomic data identifies *Non-SMC Condensin I Complex, Subunit G* (*NCAPG*) and cellular maintenance processes as major contributors to genetic variability in bovine feed efficiency. PLoS One.

[24] Lindholm-Perry AK, Kuehn LA, Oliver WT (2013). Adipose and muscle tissue gene expression of two genes (*NCAPG* and *LCORL*) located in a chromosomal region associated with cattle feed intake and gain. PLoS One.

[25] Hesse D, Radloff K, Jaschke A (2014). Hepatic trans-Golgi action coordinated by the GTPase ARFRP1 is crucial for lipoprotein lipidation and assembly. J Lipid Res.

[26] Hatzirodos N, Hummitzsch K, Irving-Rodgers HF (2014). Transcriptome profiling of granulosa cells from bovine ovarian follicles during atresia. BMC Genomics.

[27] Wadelin FR, Fulton J, Collins HM (2013). PRAME is a golgi-targeted protein that associates with the Elongin BC complex and is upregulated by interferon-gamma and bacterial PAMPs. PLoS One.

[28] Lia H, Chenga J, Wua W (2014). SRSF10 regulates alternative splicing and is required for adipocyte differentiation. Mol Cell Biol.

[29] Vernia S, Edwards YJ, Han MS (2016). An alternative splicing program promotes adipose tissue thermogenesis. Elife.

[30] Hoashi S, Hinenoya T, Tanaka A (2008). Association between fatty acid compositions and genotypes of *FABP4* and *LXR-alpha* in Japanese Black cattle. BMC Genet.

[31] Narukami T, Sasazaki S, Oyama K (2011). Effect of DNA polymorphisms related to fatty acid composition in adipose tissue of Holstein cattle. Anim Sci J.

[32] Cho S, Park TS, Yoon DH (2008). Identification of genetic polymorphisms in FABP3 and FABP4 and putative association with back fat thickness in Korean native cattle. BMB Rep.

[33] Barton L, Bures D, Kott T, Rehak D (2016). Associations of polymorphisms in bovine DGAT1, FABP4, FASN, and PPARGC1A genes with intramuscular fat content and the fatty acid composition of muscle and subcutaneous fat in Fleckvieh bulls. Meat Sci.

[34] Anton I, Kovács K, Holló G (2011). Effect of leptin, DGAT1 and TG gene polymorphisms on the intramuscular fat of Angus cattle in Hungary. Livest Sci.

[35] Koohmaraie M, Kent MP, Shackelford SD, Veiseth E, Wheele TL (2002). Meat tenderness and muscle growth: is there any relationship?. Meat Sci.

[36] Thompson JM (2004). The effect of marbling on flavour and juiciness scores of cooked beef, after adjusting to a constant tenderness. Aust J Exp Agric.

